# High-Sensitivity MXene-Functionalized Photonic Crystal Fiber Surface Plasmon Resonance Sensor with Dual Rectangular Grooves for Cancer Detection

**DOI:** 10.3390/s25185705

**Published:** 2025-09-12

**Authors:** Min Lu, Yan He, Shuyu Xi, Pufan Zhong, Yu Zhang, He Tian, Yongmei Wang, Hanglin Lu, Junhui Hu, Jian Tang

**Affiliations:** University Engineering Research Center of Advanced Functional Materials and Intelligent Sensing, College of Physics and Technology, Guangxi Normal University, Guilin 541004, China; minlu@stu.gxnu.edu.cn (M.L.); 2024012621@stu.edu.cn (Y.H.); zpf@stu.gxnu.edu.cn (P.Z.); 13982433518@163.com (Y.Z.); helynlu@gxnu.edu.cn (H.L.); hujh@mailbox.gxnu.edu.cn (J.H.)

**Keywords:** MXene, photonic crystal fiber (PCF), surface plasmon resonance (SPR), finite element method (FEM), biosensors

## Abstract

Early detection of cancer remains a key challenge because current SPR-PCF sensors lack both sensitivity and robust light–analyte interaction. To overcome these limitations, this study proposed and validated an SPR biosensor utilizing MXene-functionalized PCF. By introducing a composite structure of MXene nanomaterials and Au, the detection performance of the sensor was significantly improved. The sensor adopts a circular air hole arrangement and double-groove morphology design and leverages MXene’s high conductivity and gold’s chemical stability to simultaneously enhance plasmonic coupling and biocompatibility. Through FEM-based structural optimization of the air hole diameter, Au layer thickness, and groove shape, the sensor exhibited outstanding refractive-index detection performance with a wavelength sensitivity of 11,072 nm/RIU, an impressive quality factor reaching 201.3 RIU^−1^, and a resolution as fine as 9.03 × 10^−6^ RIU. The simulation results demonstrated the capability of the sensor to discriminate six distinct cancer-cell types (cervical cancer HeLa, leukemia Jurkat, pheochromocytoma PC-12, triple-negative breast cancer MDA-MB-231, and breast cancer MCF-7) with high sensitivity and verify its ability to detect pan-cancer species. This study demonstrates an innovative approach for constructing a high-performance SPR sensing platform that has important application potential in the context of the early detection of multiple cancers.

## 1. Introduction

Cancer, a complex multi-organ disease, remains a critical challenge for global public health. Its pathogenesis involves the malignant proliferation and metastatic dissemination of abnormal cells that evade physiological regulation, ultimately leading to lethal outcomes. Carcinogenesis arises from the interplay between genetic mutations and environmental factors, including tobacco use, air pollution, ionizing radiation, pathogenic infections, and unhealthy lifestyles (e.g., alcohol abuse and physical inactivity), all of which are established risk factors. Early diagnosis substantially increases the 5-year survival rate to over 80%, whereas advanced-stage diagnoses are linked to rates below 20% [1], highlighting the urgency of developing efficient detection technology. Despite the clinical gold-standard status of current imaging techniques (computed tomography/magnetic resonance imaging) and tissue biopsies, their limitations—including ionizing radiation, invasiveness, high equipment dependency, and prolonged turnaround time—hinder widespread adoption. These defects further aggravate delays in diagnosis and treatment, particularly in areas where medical resources are scarce [2]. To this end, researchers have turned their attention to biosensor technology, which can provide ultra-early warnings at the molecular level by specifically recognizing circulating tumor DNA (ctDNA), exosomes, or protein markers (e.g., CEA and PSA) in body fluids, such as blood and saliva [3]. Photonic crystal fiber-based surface plasmon resonance (PCF-SPR) sensors have attracted significant attention from researchers owing to their unlabeled sensing and real-time detection capabilities, as well as their structural tunability. This technology has been demonstrated in applications ranging from temperature sensing [4] to dual-parameter detection [5]. By designing the arrangement of holes in the fiber core to modulate the refractive index (RI) gradient of the cladding, the coupling between the evanescent field and target object can be enhanced.

The PCF-SPR biosensor operates through evanescent field coupling to surface plasmon polaritons (SPPs) at the metal–dielectric interface. As the guided light interacts with the interface beyond the fiber core, its oscillating electric field excites coherent electron density oscillations in the metal film, generating propagating SPP waves along the interface [6]. When the phase-matching condition is satisfied [7], light energy is transferred from the core mode to the SPP mode and there is a significant loss peak in the transmission spectrum. The wavelength associated with this condition is termed the resonance wavelength (RW) [8,9]. Analyte binding at the metal–dielectric interface induces localized RI variations that modulate the SPP propagation constant. This perturbation produces a quantifiable RW shift, enabling the precise detection of target biomolecules using surface plasmon resonance (SPR) spectroscopy. SPR sensors predominantly utilize silver, copper, and gold (Au) as plasmonic metals. Among them, Au and silver are widely used because of their excellent plasma characteristics [10,11]. Silver has extremely high sensitivity, sharp resonance peaks, and high detection accuracy; however, its chemical stability is poor and it is easily oxidized, which limits its practical application. In contrast, Au exhibits excellent biocompatibility and chemical inertness and can remain stable for a long time, especially in aqueous phases or biological environments. Therefore, they have become preferred materials in the field of biosensors [10]. In recent years, researchers have attempted to combine Au with new two-dimensional materials to enhance their performances. For example, the unique attributes of MXenes (large surface area, excellent conductivity, and modifiable surface chemistry) make them an optimal interfacial layer for improving the sensitivity and selectivity of SPR biosensors [12]. The rich surface chemistry allows for functionalization with biomolecular probes (e.g., antibodies, aptamers), which is the fundamental strategy for achieving specific cancer-cell recognition [13]. When MXene is combined with Au, the interfacial forces substantially enhance the adhesion of the Au film to the fiber substrate, thereby preventing delamination. Simultaneously, the surface functional groups of MXenes optimize the electric field distribution and enhance the local plasmon resonance effect, thereby enhancing the sensitivity of the sensor [14]. In addition, the chemical stability of Au itself enables it to resist oxidative corrosion. This characteristic makes Au-MXenes a novel SPR material with high sensitivity and stability [14].

PCF-SPR sensors have significant advantages for cancer-cell detection. Nagavel [15] proposed a dual-core PCF-SPR biosensor that could identify cancer cells early. The biosensor was designed as a double-sided polished D-shaped structure composed of hexagonally arranged circular pores. The polished area was coated with Au as the plasmonic material. The sensor exhibited wavelength sensitivities of 5714.28, 4285.72, 4285.71, 3333.33, and 3000 nm/RIU for the MCF-7, MDA-MB-231, PC-12, HeLa, Jurkat, and basal cancer cells, respectively, with a maximum resolution of 3.33 × 10^−5^ RIU. In another study, A. Kumar et al. [16] developed an ultra-sensitive photonic crystal fiber sensor with a Au-MXene coating for detecting cancer cells. The pores of the sensor were designed to be hexagonally arranged, and the Au-MXene mixed layer was annularly coated onto the photonic crystal fiber. The sensor achieves maximum wavelength sensitivities of 10,714 nm/RIU (x-polarization) and 13,071 nm/RIU (y-polarization) for MCF-7 cells, with an exceptional resolution of 1 × 10^−6^ RIU, demonstrating high-performance biosensing capabilities. Ibrahimi et al. [17] proposed a PCF-SPR biosensor with high sensitivity, air holes arranged in a circular pattern, and symmetrical C-shaped grooves on both sides of the PCF coated with graphene and Au films. Numerical evaluation shows that the sensor has the highest sensitivity to MCF-7 cells, reaching 2142.86 nm/RIU under spectral demodulation. In terms of structural innovation, Abdelghaffar et al. [18] designed a PCF biosensor based on SPR, and evaluated its potential for cancer-cell detection. Their structure features a V-shaped geometry with a ZrN coating serving as the plasmonic material. The wavelength sensitivities of breast cancer, basal carcinoma, and cervical cancer cells were 6214.28, 3800, and 5008.33 nm/RIU, respectively, in x-polarization mode. In the y-polarization mode, these values were 6000, 4400, and 5333.3 nm/RIU, respectively. R. Kumar et al. [19] introduced a novel SPR biosensor that enhanced the sensitivity of HeLa and MCF-7 cells to 4078.43 and 4285.71 nm/RIU, respectively, by employing a Au-TiO_2_ nano-mixing layer, and the substrate resolution was 4.0 × 10^−5^ RIU, confirming the effectiveness of the material composite strategy. Although the above studies have significantly improved the sensitivity of SPR sensors through material and structural optimization, there are still limitations to their application, such as the detection range covering only two specific cancer-cell types. In addition, traditional metal-oxide coatings (such as TiO_2_ and ZrN) are susceptible to oxidation and lack long-term stability, which may limit the reusability of the sensor [20]. In view of these defects, this study innovatively used a Au-MXene composite coating as a plasmonic material. The high specific surface area of MXene materials enhances the adsorption capacity of various cancer cells through abundant surface functional groups [21]. Simultaneously, the two-sided groove structure design overcomes the complex manufacturing limitations of the traditional D-type or V-type polishing process and realizes the parallel detection of multiple types of cancer markers while ensuring high sensitivity.

Despite these advancements, certain inherent limitations persist in current sensor designs. For example, while bilateral polishing techniques enable the creation of sensing platforms [15], they may introduce surface roughness that can contribute to scattering losses and spectral broadening of resonance peaks. Likewise, C-shaped groove configurations, which offer valuable symmetry [17], entail geometric curvature that could compromise mode purity and constrain achievable spectral sharpness. In response to these challenges, this work proposes an alternative approach employing a gold–MXene composite to enhance electric field intensity and bioaffinity, integrated with a symmetrically arranged dual-rectangular groove structure. The straight-wall geometry is designed to mitigate higher-order modes and reduce scattering losses, offering a potential improvement in optical performance. Moreover, the structure is compatible with high-precision femtosecond laser machining, enabling precise fabrication and efficient plasmonic coupling.

In this study, we proposed a novel dual-slot PCF sensor based on the principle of SPR for detecting multiple cancer-cell types. The sensor uses a plasmonic Au-MXene composite coating to identify six cancer-cell types: basal cells, HeLa cells, Jurkat cells, PC-12 cells, MDA-MB-231 cells, and MCF-7 cells. The performance of the sensor was confirmed through finite element method (FEM) simulations, and its resolution was evaluated to characterize the sensitivity for detecting changes in the RI of the cell. The results show that the sensor has significant potential for the rapid and accurate identification of six cancer-cell types.

## 2. Materials and Methods

### 2.1. Sensor Model and Sensing Principle

**Figure 1:**
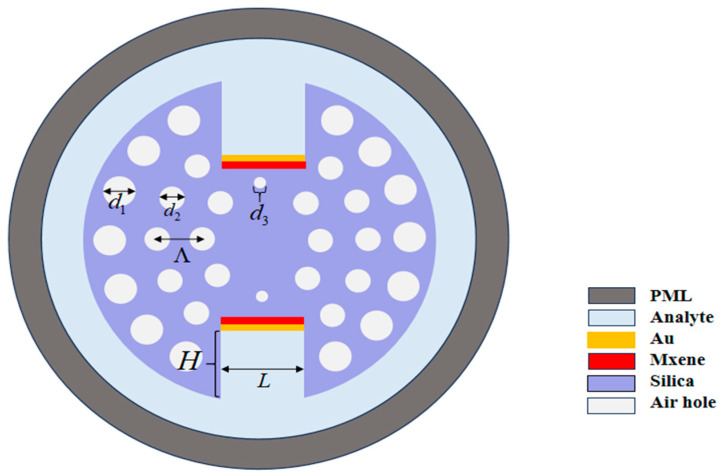
The proposed PCF-SPR biosensor.

The two-dimensional cross-sectional geometry of the proposed dual-rectangular-groove PCF-SPR sensor is shown in Figure 1.

The geometric parameters of the rectangular grooves are depth (H) = 3.7 μm and width (L) = 3.6 μm. The fiber core featured a triple-layer annular air-hole arrangement at azimuthal angles of 18°, 22.5°, and 45°. A highly efficient light-field localization was achieved by accurately adjusting the pore diameter. The outermost layer pore diameter d1 = 1.3 μm, the middle layer pore diameter d2 = 0.9 μm, and the core region form an RI gradient by replacing the upper and lower symmetrical position pore diameter d3 = 0.45 μm. The spacing of each pore layer is Λ = 2 μm, and the parameter design considers both mode constraints and energy coupling efficiency. A Au-Ti3C2Tx composite layer (Au thickness tg = 40 nm and MXene thickness tm = 14 nm) was coated onto the surface of the square groove. The MXene layer functioned as a dielectric medium between silica and Au, thereby enhancing the SPR generation at the metal–dielectric interface. This enhancement facilitates stronger molecular interactions and plasmon resonance, allowing the sensor to detect minute changes in the RI [22]. To minimize boundary reflection artifacts in FEM simulations, a 1.2 μm thick perfectly matched layer (PML) encloses the computational domain.

The sensor substrate is composed of fused silica, with its wavelength-dependent RI governed by the Sellmeier dispersion relation [23].

(1) $$n_{silica}=\sqrt{1+\frac{A_{1}\lambda^{2}}{\lambda^{2}-B_{1}}+\frac{A_{2}\lambda^{2}}{\lambda^{2}-B_{2}}+\frac{A_{3}\lambda^{2}}{\lambda^{2}-B_{3}}}$$

The coefficients of the Sellmeier equation are *A*_1_ = 0.696163, *A*_2_ = 0.4079426, *A*_3_ = 0.8974794, *B*_1_ = 0.0046791486, *B*_2_ = 0.0135120631, and *B*_3_ = 97.934003. In Equation (1), λ represents the optical wavelength. The complex dielectric constant of Au is described by the Drude–Lorentz model [24]:

(2) $$\varepsilon_{Au}=\varepsilon_{\infty }-\frac{\omega_{D}^{2}}{\omega \left(\omega +j\gamma_{D}\right)}-\frac{\Delta \varepsilon \;\Omega_{L}^{2}}{\omega \left(\omega -\Omega_{L}^{2}\right)+j\Gamma_{L}\omega }$$

where $\varepsilon_{Au}$ represents the dielectric constant of Au, and $\varepsilon_{\infty }=5.9673$ represents the high-frequency permittivity [25]. The angular frequency  ω is given as ω=2πc/λ, where *c* is the speed of light. The plasma frequency $\omega_{D}$ and damping frequency $\gamma_{D}$ are defined as $\omega_{D}=2113.6\times 2\pi$ THz and $\gamma_{D}=15.92\times 2\pi$ THz, respectively. The weighting factor Δε is 1.08. The Lorentz oscillator strength $\Omega_{L}$ and spectral width $\Gamma_{L}$ are given as $\Omega_{L}=650.07\times 2\pi \;\mathrm{THz}$ and $\Gamma_{L}=104.86\times 2\pi \;\mathrm{THz}$, respectively [26].

### 2.2. Sensor Fabrication and Materials

The sensor can be fabricated using the ‘stack-and-draw technique’ to prepare the PCF preforms. This method can perfectly reproduce the complex pore structure by accurately controlling the capillary arrangement and stretching temperature. A rectangular groove structure can be symmetrically etched at the top and bottom of the fiber using femtosecond-laser micromachining technology [27]. A metal functional layer can be deposited using magnetron sputtering combined with the chemical vapor deposition method. Several common techniques may be employed to deposit Au and MXene layers on the surface of PCF, with thermal evaporation and RF sputtering being the primary methods.

MXenes represent a novel class of two-dimensional transition metal carbides/nitrides with the general chemical formula M_n+1_X_n_T_x_, where M denotes a transition metal, X is C or N, and T_x_ indicates surface functional groups (–O, –OH, –F).

The Ti_3_C_2_T_x_ variant, for instance, is synthesized through the selective etching of aluminum layers from its parent MAX phase (Ti_3_AlC_2_), resulting in a characteristic accordion-like layered morphology. The abundant functional groups on the surface of MXene endow it with excellent hydrophilicity, chemical activity, high intrinsic conductivity (103–104 S/cm), and mechanical flexibility, rendering it a suitable candidate for photoelectric sensing applications [28]. The literature reports [13] indicate that Ti_3_C_2_T_x_ cytotoxicity was at a concentration of 95%, confirming its biocompatibility.

## 3. Results and Discussion

### 3.1. Sensor Performance

**Figure 2:**
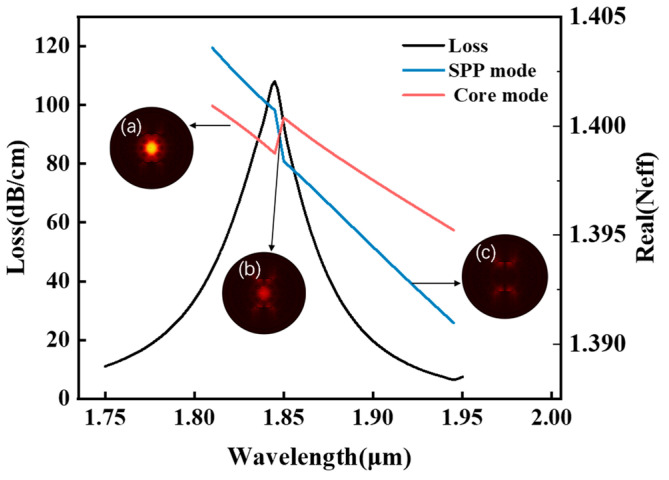
Dispersion relationship and optical field distributions between core mode and SPP mode for basal cancer cells. (**a**) Fundamental mode field distribution (RI = 1.38); (**b**) coupled hybrid mode; (**c**) SPP mode. Black curve: core mode confinement loss (left axis); Red/blue curves: real part of effective refractive indices for core mode and SPP mode, respectively (right axis).

The proposed plasmonic PCF biosensor achieved specific recognition of various cancer types by monitoring the dynamic changes in the single-cell horizontal RI. Different cancer types are closely associated with the RI characteristics of specific cell lines. Skin cancer is typically associated with basal cell characteristics, whereas breast cancer is frequently studied using cell lines such as MCF-7, MDA-MB-231, and Jurkat cells. The sensor simulation demonstrates theoretical potential by detecting the difference in the RI between cancerous and healthy cells [29], as shown in Table 1. In terms of performance evaluation, the core parameters of the sensor include constraint loss (CL), wavelength sensitivity (WS) and sensor resolution (RS). The design of the Au-MXene composite structure significantly improved sensitivity, and the maximum WS can reach 20,000 nm/RIU. The FEM simulations numerically solved Maxwell’s equations to determine the effective RI of the sensor, providing a critical insight into their optical response characteristics. The real component of the propagation constant governs the propagation behavior of the guided modes, including the core and SPP modes, whereas the imaginary component is used to quantify the associated loss characteristics. When RI reaches 1.38, the optical field limitation of the core mode is shown in Figure 2a. Under this phase-matching condition, the real effective indices of the core and SPP modes converge, enabling efficient energy transfer at the Au-MXene/silica interface through resonant plasmonic coupling. The dominant energy-loss mechanisms arise from plasmonic coupling at the metal–dielectric interface and core-mode dissipation, both of which are quantifiable through confinement loss analysis. This is expressed [30] as follows:

(3) Loss(dBcm)≅8.686×2πλ×Im(Neff)×104

**Figure 3:**
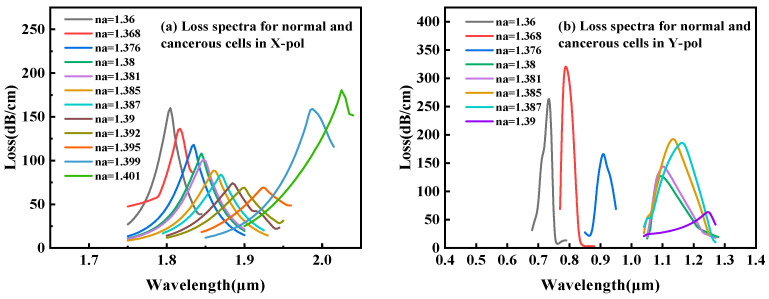
Confinement loss spectra for (**a**) x-polarized and (**b**) y-polarized modes across varying analyte refractive indices.

Following a comparative evaluation, the x-polarization mode was selected for cancer-cell detection. As illustrated in Figure 3, despite the y-polarization’s larger resonance shift (and thus higher theoretical sensitivity) for refractive indices (RI) below 1.39, its operational range is limited to an RI of 1.36–1.39—a trade-off between ultra-high sensitivity and broad detection range in SPR sensing. This narrow range only permits the detection of basal and Jurkat cells. In contrast, the x-polarization mode provides a stable, dynamic response across the comprehensive biological RI range of 1.36–1.401 RIU, enabling robust detection of all six cancer-cell types. The dispersion relationship and loss characteristics of the base cancer-cell sensor in x-polarization mode is shown in Figure 2. In the figure, the right *y*-axis corresponds to the real effective RI, and the left *y*-axis indicates the loss (*Loss*). Figure 2 also shows the spectral characteristics of the sensor through three distinct traces: the black curve displays the wavelength-dependent core mode confinement loss across the 1.750–1.950 μm range, whereas the red and blue curves map the dispersion of the real effective indices for the core and SPP modes, respectively. A critical phase-matching condition occurs at 1.845 μm where the confinement loss peak was aligned with the convergence of the core and SPP mode effective indices. The wavelength corresponding to this intersection is the RW, which indicates the optimal energy transfer efficiency between the core guiding mode and SPP mode. Figure 2a–c present the optical field distributions for the (a) fundamental, (b) coupled hybrid, and (c) SPP modes.

**Figure 4:**
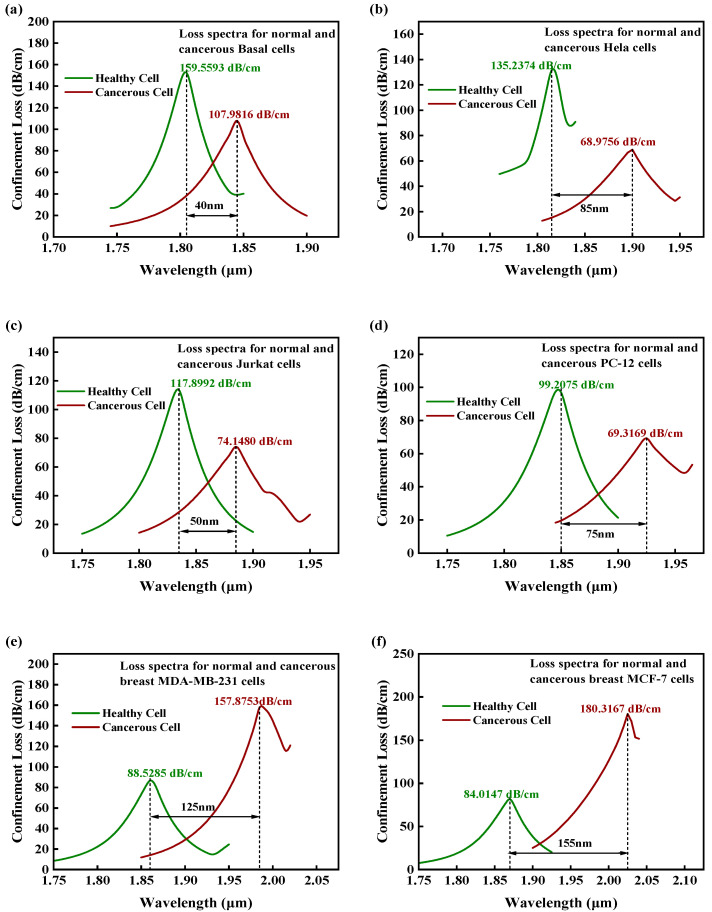
Dispersion relation with loss spectra of core mode and surface plasmon polariton mode for healthy and cancerous cells in x-polarization mode: (**a**) basal cells, (**b**) HeLa cells, (**c**) Jurkat cells, (**d**) PC-12 cells, (**e**) MDA-MB-231 cells, and (**f**) MCF-7 cells. Green curves: healthy cells; red curves: cancer cells (exhibiting significant red shifts Δλ = 40–155 nm).

Figure 4a–f show the simulated loss spectra of somatic cells in both the physiological and malignant states. Spectral signatures revealed distinct profiles between healthy (green curve) and cancerous (red curve) cell populations. The comparative analysis shows that compared with healthy cells, the resonance loss peaks of all types of cancer-cell samples show a significant red shift (wavelength increment range: ∆λ = 40−155 nm). Through precise phase matching between the incident light and SPW, a significant RW shift is induced. In terms of sensitivity evaluation, this study uses the wavelength modulation method to quantitatively characterize the sensing performance by monitoring the linear relationship between the RW offset (Δλ) and the RI change (Δn). The wavelength sensitivity WS based on this method is calculated as follows [19]:

(4) $$S_{\lambda }\left(\mathrm{nm}/\mathrm{RIU}\right)=\frac{\Delta \lambda_{peak}}{\Delta n_{a}}$$

where $S_{\lambda }$ is the WS and $\Delta n_{a}$ is the difference in RI between healthy cells and cancer cells. As shown in Figure 4a–f, the sensor had a minimum WS of 2000 nm/RIU for basal cells and a maximum WS of 11,072 nm/RIU for MCF-7 breast cancer cells. Resolution, representing the minimum detectable RI change, serves as the fundamental parameter for determining biosensor detection limits, which are defined as the minimum RI change or cell concentration difference that the sensor can stably identify. The formula is as follows [31]:

(5) $$R_{s}=\Delta n_{a}\frac{\Delta \lambda_{\min}}{\Delta \lambda_{peak}}$$

where $\Delta \lambda_{min}$ is the minimum detectable RI change under the limitation of system noise. In a sensor performance-evaluation system, in addition to sensitivity and resolution, the figure of merit (FOM) serves as a crucial performance metric for evaluating the overall sensing capabilities of the device. This parameter is expressed in terms of WS and the full width at half maximum (FWHM) of the resonance peak; it is mathematically expressed as [32]

(6) $$FOM=\frac{S_{\lambda }}{FWHM}$$

**Figure 5:**
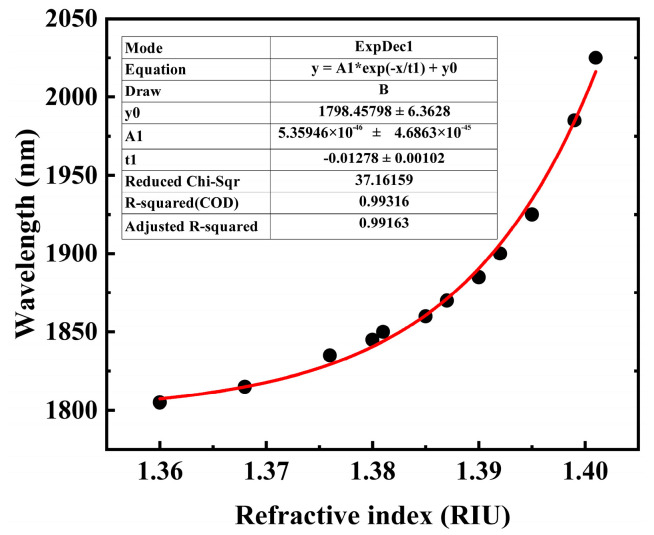
Sensor calibration curve and sensitivity fitting. *X*-axis: cell refractive index (1.36–1.401 RIU); *Y*-axis: resonance wavelength (RW). Black dots: simulation data; red line: linear regression fit (R^2^ = 0.99316).

A higher FOM value indicates that the sensor achieves high sensitivity while maintaining a narrow resonance peak width with notable detection precision and signal-to-noise ratio. Table 2 summarizes the key performance metrics of the proposed sensor. The simulation results for different cancer-cell types in this study showed that the FOM value of the MCF-7 cell detection unit reaches 201.3 RIU^−1^, which is significantly higher than that of similar SPR sensors. This advantage is due to the optimized two-sided groove structure and the MXene plasmon enhancement effect, which compresses the FWHM to 55 nm while maintaining high sensitivity (WS = 11,072 nm/RIU). Figure 5 summarizes the calibration across 1.36–1.401 RIU, confirming the sensor’s quantitative reliability prior to cancer-cell assays.

The proposed sensor operates in the 1.75–2.10 µm range, which contains a strong water absorption band [33]. Crucially, the ultrashort penetration depth (<1 µm) of the SPR evanescent field naturally mitigates this issue by minimizing the interaction volume with water [34]. For practical implementation, suitable optical components, such as extended InGaAs photodiodes and superluminescent diodes, are readily available for this wavelength window [35].

### 3.2. Optimization of Geometric Parameters

The structural parameters of the PCF-based sensor, including the pore dimensions, air hole pitch, Au film thickness, and depth of the square groove, critically governed its performance by mediating the incident light–plasmon coupling efficiency. To identify the optimal configurations of these parameters, we systematically analyzed the changes in the coupling loss (CL) and WS.

**Figure 6:**
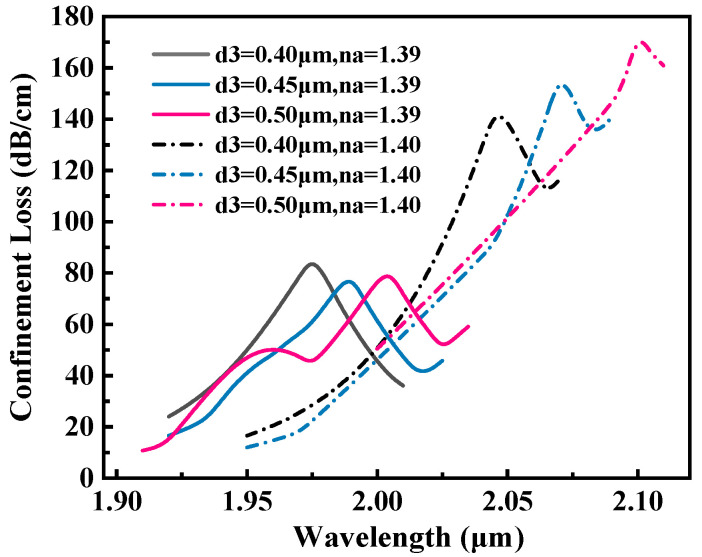
Loss spectra of the proposed sensor at RI of 1.39 and 1.40 for $d_{3}$ = 0.4, 0.45, and 0.50 μm.

Figure 6 illustrates the effect of varying small pore diameters $\left(d_{3}=0.40,0.45,0.50\mu m\right)$ on RW shift behavior of the sensor. The simulation results show that with the increase in $\;d_{3}$ from 0.40 μm to 0.50 μm, the observed redshift in the RW is consistent with the mechanism by which an increase in pore size reduces the effective RI of the core mode and induces a redshift in the coupling wavelength. Specifically, when $d_{3}$ increases from 0.40 μm to 0.45 μm, the observed increase in RW from 70 nm to 80 nm directly correlates with an enhanced sensitivity of the sensor.

This phenomenon is primarily caused by the expansion of the pore sizes, which promotes a stronger interaction between the evanescent field and the analyte, thereby enhancing the plasmonic energy coupling efficiency. However, when the $d_{3}$ increases further to 0.50 μm and the RI of analyte n is 1.39 (red curve), although the RW shift continues to increase to 95 nm, the loss peak is obviously distorted, as evidenced by the broadening of the FWHM. This phenomenon may be related to the decrease in the coupling efficiency between the SPP and core modes caused by the large diameter of the small pores. An increase in the FWHM directly reduces the quality factor of the sensor. Considering the key parameters such as sensitivity, FWHM, and FOM, $d_{3}\;=\;0.45\;\mu m$ is selected as the optimal value. The corresponding resonance peak is sharp and the offset is moderate, which can achieve better balance between high sensitivity and low noise.

**Figure 7:**
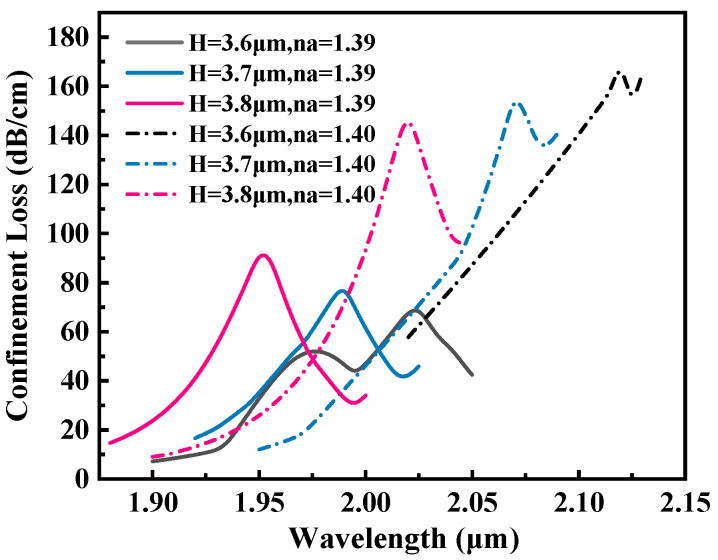
Loss spectra of the proposed sensor at RI of 1.39 and 1.40 for *H* = 3.6, 3.7, and 3.8 μm.

The groove depth is the core structural parameter, and its optimization has a regulatory effect on the efficiency of the sensor. Figure 7 shows the influence of different groove depths (H=3.6,3.7,3.8μm) on the RW shift characteristics of the sensor. The simulation results show that as H increases from 3.6μm to 3.8μm, the RW exhibits a blue-shift pattern, accompanied by an increasing offset. Similarly, when H increases to 3.8μm and the RI of the analyte is 1.39 (black curve), the loss peak also appears with obvious distortion. Considering the key parameters such as sensitivity, FWHM, and FOM, H=3.7μm is selected as the optimal value.

**Figure 8:**
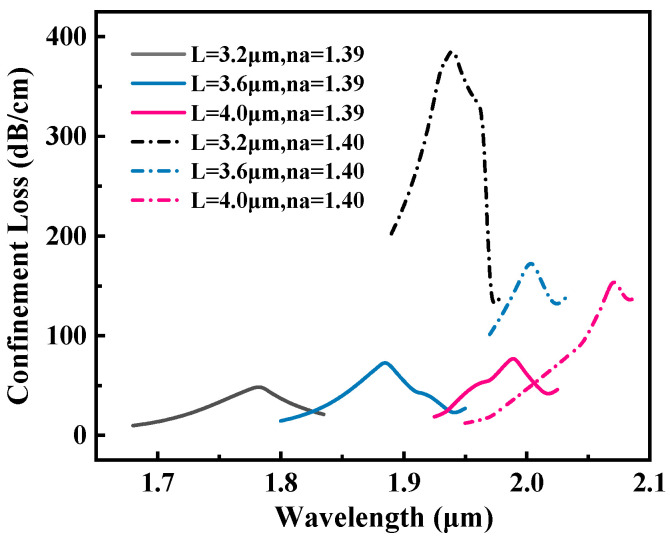
Loss spectra of the proposed sensor at RI of 1.39 and 1.40 for *L* = 3.2, 3.6, and 4.0 μm.

The width of the square groove also affected the sensing efficiency of the biosensor. Figure 8 shows the influence of different groove widths (L=3.2,3.6,4.0μm) on the performance of the sensor. As the square slot width decreases from 4.0μm to 3.2μm, the RW shift increases. However, when L=3.2μm, although the sensitivity is the highest $\left(WS=15,500\;\mathrm{nm}/\mathrm{RIU}\right)$, the loss value at n=1.40 increases sharply to 390 dB/cm, which is about twice the loss at L=3.6 μm. Considering the sensitivity and reliability, L=3.6 μm is selected as the optimal value.

**Figure 9:**
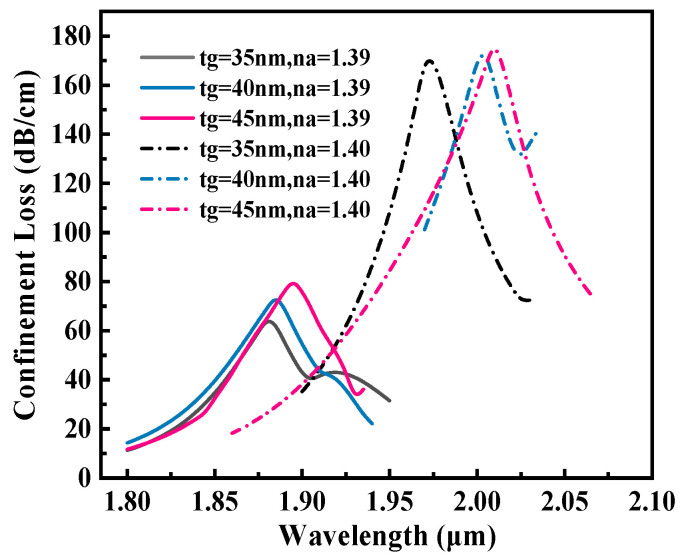
Loss spectra at RI of 1.39 and 1.40 for various gold thicknesses t_g_ = 35, 40, and 45 nm.

Figure 9 shows the influence of different Au layer thicknesses (tg = 35, 40, 45 nm) on the RW shift characteristics of the sensor. The simulation data show that when the thickness of the Au layer is 40 nm, the sensor demonstrates optimal performance within the RI range of 1.39–1.40, achieving a peak RW shift and maximum sensitivity of 12,000 nm/RIU. The physical mechanism behind this result is that when tg = 35 nm, the Au layer is too thin to cause the evanescent field to penetrate excessively into the analyte, which weakens the local field enhancement effect of the SPP. At tg = 45 nm, the Au layer was too thick to allow the light energy to be strongly absorbed by the metal, and the SPR coupling efficiency was significantly reduced. Therefore, tg = 40 nm is selected as the optimal value.

The proposed design exhibits high tolerance to fabrication variances. FEM simulations show that within practical tolerances (±5 nm for Au, ±0.1 µm for groove), the sensor’s performance consistently remains in the high-sensitivity regime of 9000–12,000 nm/RIU. Notably, even in the worst-case scenario, the sensitivity remains quite competitive, ensuring reliable detection capability without requiring extreme fabrication precision.

Based on simulation data, we calibrated the sensor across the 1.36–1.401 RIU range, which encompasses both physiological and cancerous cell refractive indices. The resulting linear relationship (R^2^ = 0.993) confirms its quantitative capability within this biologically relevant window, laying the groundwork for reliable cancer-cell identification.

Table 3 presents a detailed performance comparison of the proposed biosensor and recently reported PCF-SPR sensors for cancer detection. The proposed biosensor design has a compact design, external sensing functionality, and a novel two-layer plasmonic architecture. Compared to previously developed cancer-detection sensors, the proposed biosensor exhibited commendable sensing parameters. Thus, this biosensor could differentiate healthy cells from cancerous cells and serve as a reliable clinical diagnostic tool for cancer detection.

## 4. Conclusions

In this study, a novel PCF-SPR biosensor was designed and evaluated through FEM simulations. The sensitivity and specificity of the sensor were significantly enhanced by introducing a symmetrical dual-rectangular groove structure. Using circularly arranged air holes, we constructed a composite structure with gold as the plasmonic excitation layer and MXene as the field-enhancing interlayer, which enhanced the coupling between the evanescent field and the analyte. Through FEM optimization of structural parameters (air hole diameter, Au layer thickness, and slot geometry), the sensor demonstrated exceptional performance in key metrics: exhibiting a sensitivity of 11,072 nm/RIU, with an FOM reaching 201.3 RIU^−1^ and a detection resolution of 9.03 × 10^−6^ RIU. The simulation results confirmed the robust response of the sensor to six cancer-cell types: basal cells, cervical cancer cells (HeLa), T-lymphocytic leukemia cells (Jurkat), pheochromocytoma cells (PC-12), and the breast cancer cells MDA-MB-231 and MCF-7.

## Figures and Tables

**Table 1:** Refractive index of normal and cancer cells.

Cell Name	Cancer Type	RI Normal Cells	RI Cancer Cells	∆RI
Basal	Skin	1.360	1.380	0.020
Hela	Cervical	1.368	1.392	0.024
Jurkat	Blood	1.376	1.390	0.014
PC-12	Adrenal gland	1.381	1.395	0.014
MDA-MB-231	Breast type-2	1.385	1.399	0.014
MCF-7	Breast type-1	1.387	1.401	0.014

**Table 2:** Detection performance metrics of the proposed biosensor across multiple cancer-cell types.

Cell Name	RW Shift (nm)	WS (nm/RIU)	*R_S_* (RIU)	FWHM (nm)	FOM (RIU^−1^)
Basal	40	2000	5 × 10^−5^	35	57.14
Hela	85	3542	2.82 × 10^−5^	30	118.06
Jurkat	50	3572	2.8 × 10^−5^	40	89.30
PC-12	75	5357	1.86 × 10^−5^	50	107.14
MDA-MB-231	125	8929	1.12 × 10^−5^	50	178.58
MCF-7	155	11,072	9.03 × 10^−6^	55	201.30

**Table 3:** Performance comparison.

Sensor	Cell Name	WS (nm/RIU)	Resolution (RIU)	Ref.
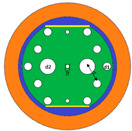	Basal	3000	3.33 × 10^−5^	[15]
	Hela	3333.33	3 × 10^−5^	
	Jurkat	4285.72	2.33 × 10^−5^	
	PC-12	4285.72	2.33 × 10^−5^	
	MDA-MB-231	5714.28	1.75 × 10^−5^	
	MCF-7	5714.28	1.75 × 10^−5^	
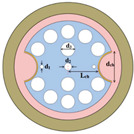	Basal	1500	6.7 × 10^−5^	[17]
	Hela	1666.67	6 × 10^−5^	
	Jurkat	1428.57	7 × 10^−5^	
	PC-12	1428.57	7 × 10^−5^	
	MDA-MB-231	2142.86	4.67 × 10^−5^	
	MCF-7	2142.86	4.67 × 10^−5^	
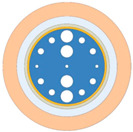	Basal	2500	4 × 10^−5^	[19]
	Hela	2916.66	3.42 × 10^−5^	
	Jurkat	3571.42	2.8 × 10^−5^	
	PC-12	3571.42	2.8 × 10^−5^	
	MDA-MB-231	4285.71	2.33 × 10^−5^	
	MCF-7	4285.71	2.33 × 10^−5^	
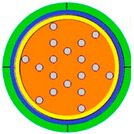	Basal		2 × 10^−5^	[36]
	Hela		1.5 × 10^−5^	
	Jurkat		1.4 × 10^−4^	
	PC-12		1.4 × 10^−4^	
	MDA-MB-231		2.33 × 10^−4^	
	MCF-7		2.33 × 10^−4^	
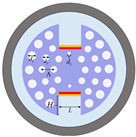	Basal	2000	5 × 10^−5^	This Work
	Hela	3542	2.82 × 10^−5^	
	Jurkat	3572	2.8 × 10^−5^	
	PC-12	5357	1.86 × 10^−5^	
	MDA-MB-231	8929	1.12 × 10^−5^	
	MCF-7	11,072	9.03 × 10^−6^	

## Data Availability

The data supporting this study’s findings are included in the article.
